# Kyphoplasty in osteoporotic vertebral compression fractures - Guidelines and technical considerations

**DOI:** 10.1186/1749-799X-6-43

**Published:** 2011-08-19

**Authors:** Yohan Robinson, Christoph E Heyde, Peter Försth, Claes Olerud

**Affiliations:** 1 Uppsala University Hospital, Institute for Surgical Sciences, Department of Orthopaedics, Uppsala, Sweden; 2 Leipzig University Hospital, Department of Orthopaedic Surgery, Spine Surgery, Leipzig, Germany

**Keywords:** Kyphoplasty, vertebroplasty, osteoporosis, spinal fractures

## Abstract

Osteoporotic vertebral compression fractures are a menace to the elderly generation causing diminished quality of life due to pain and deformity. At first, conservative treatment still is the method of choice. In case of resulting deformity, sintering and persistent pain vertebral cement augmentation techniques today are widely used. Open correction of resulting deformity by different types of osteotomies addresses sagittal balance, but has comparably high morbidity.

Besides conventional vertebral cement augmentation techniques balloon kyphoplasty has become a popular tool to address painful thoracic and lumbar compression fractures. It showed improved pain reduction and lower complication rates compared to standard vertebroplasty. Interestingly the results of two placebo-controlled vertebroplasty studies question the value of cement augmentation, if compared to a sham operation. Even though there exists now favourable data for kyphoplasty from one randomised controlled trial, the absence of a sham group leaves the placebo effect unaddressed. Technically kyphoplasty can be performed with a transpedicular or extrapedicular access. Polymethyl methacrylate (PMMA)-cement should be favoured, since calcium phosphate cement showed inferior biomechanical properties and less effect on pain reduction especially in less stable burst fractures. Common complications of kyphoplasty are cement leakage and adjacent segment fractures. Rare complications are toxic PMMA-monomer reactions, cement embolisation, and infection.

## Introduction

Osteoporosis and pathological osteoporotic fractures are common findings in the elderly population. The age-standardised annual incidence of vertebral compression fractures (VCF) is 10.7/1000 in women and 5.7/1000 in men, increasing markedly with age [1]. At the age of 75 to 79 the annual incidence was 29.3/1000 in women and 13.6/1000 in men. Due to the continued aging of our population, VCF represent a major cause of disability and are a burden to the national healthcare budgets [2]. Non-surgical management with pain control and physical therapy-assisted mobilization has for a long time been the only treatment option in VCF. Unfortunatelty a great number of patients remain functionally impaired after VCF, and some of them are severely handicapped due to chronic back pain [3]. The functional and physical consequences of VCF lead to anxiety, depression, and have devastating impact on interpersonal relationships and social roles [4]. It is therefore no surprise that untreated VCF contribute significantly to shorter life-expectancy both in women (mortality ratio 1.66, p < 0.01) and even greater in men (mortality ratio 2.38, p < 0.0001) within one year after onset of symptoms [5].

### Indications for cement augmentation

while medical therapy of osteoporosis improves dramatically, the restoration of quality of life is still a major issue in VCF treatment. Osteoporotic kyphotic compression fractures often lead to a anterior shift of the sagittal plumb line and increased load of the anterior vertebral column, which may cause further compression fractures [6]. This cascade of sequential compression fractures is eventually causing the typical hump of the elderly, with significant thoracic kyphosis and low pelvic incidence, forcing the patient to bend hips and knees to maintain sagittal balance [7].

Galibert et al [8] presented the first cases of successful vertebral augmentation by intravertebral injection (vertebroplasty) of polymethyl methacrylate (PMMA) in patients with vertebral haemagiomas. Later, vertebroplasty was successfully introduced for the management of osteoporotic compression fractures [9]. The primary goal of vertebroplasty is pain relief by stabilization of the VCF, improving indirectly pulmonary function and patient quality of life [10]. The biomechanical understanding of increasing anterior column load with progressing kyphosis leading to subsequent VCF established the basic rationale for kyphoplasty. With this technique, partial reduction of VCF is possible by transpedicular intracorporal balloon expansion and retention by PMMA cement augmentation [11,12]. The results of one multicenter randomised controlled trial found shortened and improved functional recovery after kyphoplasty with a low rate of complications if compared to non-surgical treatment [13].

Despite the advances in percutaneous augmentation techniques the conservative medical therapy cannot be replaced. VCF without initial kyphosis, no consecutive sintering and a satisfactory and quick response to conservative treatment should be treated conservatively. Furthermore, since lack of reimbursement in most countries kyphoplasty causes an economic burden, many patients are not willing to take. Beyond that, it has to me emphasised, that it remains unclear whether the benefits of kyphoplasty outweigh its complications.

Two placebo-controlled vertebroplasty-studies have sobering results with regard to pain and functional outcome after cement augmentation with vertebroplasty, if compared to a sham-operation [14,15]. In both studies the sham procedure included percutaneus needle insertion and opening of PMMA-monomer mixture to simulate the specific odour. The sham-controlled trial by Buchbinder et al [14] in 78 patients with MRI-confirmed, fresh and painful VCF found no beneficial effect of vertebroplasty when compared to a sham procedure. A very similar study by Kallmes et al [15] investigating 131 patients found similar results. This study had already after 3 month significant higher cross-over of 43% in the control-group (p < 0.001) diminishing the quality of this study. Furthermore only outpatients were included in this study, which means that no patients being hospitalised due to acute VCF entered the study. The randomised controlled trial by Rousing et al [16] found no greater improvement in back pain in patients treated with vertebroplasty when compared to medical therapy. Interestingly they found a significant improvement in the Barthel-score after 12 month (p < 0.02) indicating improved function, [17]. As a result to the above-mentioned results several authors abandoned the use of vertebroplasty [18-20] while others are hesitant and question the quality of the sham-controlled vertebroplasty trials [17,21]. It is unclear whether the results of the multicenter randomised kyphoplasty trial could be reproduced if sham-controlled [13]. In table 1 the authors present clinically proven guidelines for indications and contraindications of kyphoplasty.

**Table 1:** Guidelines for indications and contraindications for kyphoplasty

**Indications for kyphoplasty**	- **Radiologically confirmed fresh compression fracture (AO type A1) **(MRI shows oedema or X-ray/CT-scan proven fracture not older than 3 months)
	- **Failure of 2 - 6 weeks of conservative treatment **including pain medication and physiotherapy (Pain on visual analogous scale (VAS) above 4 of 10)
**Contraindications for kyphoplasty**	- Burst-fractures (in some A3.1-fractures possible)
	- Flexion-/distraction and rotational injuries (AO type B and C)
	- Medical contraindications (bleeding disorders, sepsis, etc)
	- PMMA-allergy

Due to the increased demand in cement augmentation techniques, procedures similar to kyphoplasty have been developed. One competitor is Vesselplasty^® ^(A-Spine), where a porous balloon is inflated within the fractured vertebral body and filled with cement without removing the balloon, thus reducing the risk of cement leakage [22]. Another new product is the Sky^® ^bone expander (Disc-O-Tech), an expandable polymer bone tamp abandoning the use of cement, which had favourable results in clinical case series [23]. Then there is the StaXx^® ^FX system (Spine Wave) where a VCF is reduced percutaneously by gradual insertion of stacked PEEK-chips into the vertebral body to reduce and stabilise the fracture [24].

### Indications for combined cement augmentation and posterior instrumentation

Lately kyphoplasty has been discussed as an alternative therapy even of burst fractures in elderly patients. This is especially true in case of AO type A3.1 fractures, where it could be applied instead of a posterior-only or 360 degrees stabilisation [25]. In many of these cases further sintering of the fractured vertebra with posterior dislocation of an instable fragment with spinal stenosis is a feared complication [26,27]. Thus several surgeons perform posterior instrumentation of the adjacent vertebrae to protect the posterior wall and to improve the sagittal profile [28]. This can be done using percutaneous posterior instrumentation or with a conventional open technique [28-30]. Possible disadvantages of this technique are due to segmental fusion an increased load of the adjacent segments with degeneration, and possible implant loosening with loss of correction due to low bone quality. Cement augmentation of the implanted pedicle screws can reduce the complication rate regarding the latter mentioned problem [31].

### Limitations of kyphoplasty and indications for open reduction and stabilisation

If multiple VCF lead to kyphosis with fixed sagittal imbalance, cement augmentation will address the fracture pain but not global imbalance. Major spinal imbalance can be a cause of significant functional disability leading to reduced quality of life. Increased kyphosis may additionally cause subsequent VCF due to an increased anterior load [32]. The anterior location of the sagittal plumb line in fixed sagittal imbalance will lead to falls with possible further fractures and morbidity. Therefore the indication for correction of global sagittal imbalance may be given in severe cases. As both open and closing wedge procedures are associated with complications leading to disabling morbidity surgeon are often hesitant to perform these operations in patients with multiple comorbidities [33]. Due to the osteopenic bone quality often long instrumentations are required. Unfortunately these an increased risk of adjacent VCF and pedicle fractures [34]. With regard to open sagittal corrections there is growing evidence that the posterior-only pedicle subtraction osteotomy is superior to multiple Smith-Petersen osteotomies, allowing greater correction with lesser operation time [35,36].

## Operation technique of kyphoplasty

### Percutaneous bilateral transpedicular kyphoplasty

**Figure 1:**
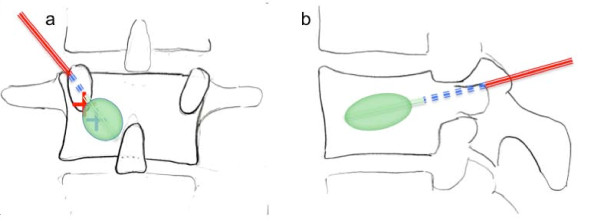
**Transpedicular approach for balloon kyphoplasty**. After entry in the craniolateral pedicle (red cross) in the p-a-projection **(a)**, the medial cortex of the pedicle is first breached when the vertebral body is entered in the lateral projection (blue cross) **(b)**. After preparation of the working channel a balloon can be placed in the vertebral body.

The bilateral transpedicular approach is the standard kyphoplasty access for the thoracolumbar spine, enabling a symmetric reposition and augmentation of the VCF. Firstly, the fracture is reduced under fluoroscopic control by positioning and traction. Then biopsy needles are used to enter the fractured vertebra on both sides through the pedicle (figure 1). If fluoroscopy confirms correct transpedicular placement of the needles in both planes, a K-wire is placed through the Jamshidi biopsy needle into the vertebral body close to the anterior wall. Then over the K-wire the access is widened with the osteointroducer. Then empty bone-fillers are used to form a cavity for the safe placement of the balloons. Under fluoroscopic control two balloons are positioned anteriorly within the vertebral body, and then the balloons are inflated under manometric control. In fresh fractures up to 150 psi balloon pressure are mostly enough, but sometimes up to 300 psi are necessary to reduce a partially healed compression fracture. One has to be careful not to fracture the endplates or the posterior wall, which could lead to cement leakage. After successful reduction the cavity is filled with bone-cement from both pedicles. This step has to be performed with careful fluoroscopic control. Normally the cement should have a tooth-paste-like viscosity and should not stick to the surgeons' gloves when testing the viscosity. Finally the introducers are removed.

### Percutaneous unilateral extrapedicular kyphoplasty

**Figure 2:**
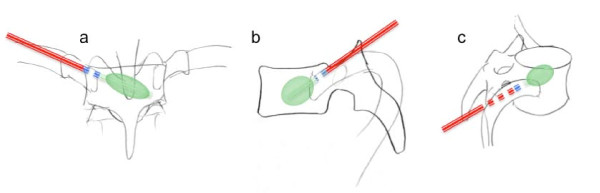
**Unilateral extrapedicular costotransversary approach for balloon kyphoplasty**. Following the cranioposterior part of the respective rib into the costotransversary space **(c) **allows extrapedicular access to the vertebral body in the thoracic spine. Due to the far lateral approach a single balloon is placed in the middle of the vertebral body **(a, b)**.

Due to the anatomical characteristics of the thoracic spine central placement of the balloons can be difficult. Firstly, the introducer often does not fit in the narrow pedicles of the thoracic spine. Then the low angulation of the pedicles does not allow a central placement of the balloons disabling adequate reduction in some cases. Thus in the thoracic spine extrapedicular accesses gain increasing popularity, avoiding pedicle perforation with possible neurological damage or intraspinal cement leakage. Most surgeon prefer the transcostovertebral access from far lateral (figure 2), guided to the collum costae into the costotransversary space to the cranio-posterior wall of the fractured vertebra, the Jamshidi needle with the tip just penetrating the lateral pedicle at its base [37,38]. In the view from posterior the needle passes above of the transverse process and meets the pedicle at the craniolateral circumference. The lateral view confirms the placement of the tip of the needle close to the base of the pedicle. In an axial view the needle is seen to pass through the costovertebral gap, between the neck of the rib and the lateral pedicle circumference, towards the base of the pedicle. Then the posterolateral cortical wall is opened with a Jamshidi cannula and widened as described above with K-wire and osteo-introducer. To allow central placement of the balloon in the vertebral body a greater angle than in the transpedicular placement has to be sought. This often requires a 7 to 10 cm off-midline percutaneus approach. A single balloon is then used for reduction and the cavity is then filled with cement as described above.

### Open unilateral interlaminary kyphoplasty

Open interlaminary kyphoplasty should be reserved for cases where an open approach has to be performed to decompress neurological structures, and the spinal canal has to be accessed anyway [39]. After open decompression the dural sac is retracted medially and the posterior wall of the fractured vertebra exposed. Now kyphoplasty can be performed with a single balloon positioned under fluoroscopical guidance in the centre of the vertebral body. After kyphoplasty the spinal canal has to be investigated for cement leakage. This method must be restricted to levels below the conus medullaris to avoid myelopathy due to manipulation within the spinal canal.

### Open anterior kyphoplasty

In rare cases kyphoplasty may be performed using an anterior access, too [40]. Through a minimally-invasive anterior access the biopsy needle may be placed directly on the anterior wall of the fractured vertebra and a single balloon be placed into the vertebral body. Then under fluoroscopical control the fracture is reduced and cement is applied.

### Technical considerations

#### Operation room setup

Both general an local anesthesia have been successfully applied for the procedure [41], but many surgeons favour general anestesia allowing closed reduction in a relaxed patient. By patient positioning only, more than 70% of vertebral height restoration can be achieved. Placing the patient in prone position lordosating the fractured segment by pillows or by bending the table will lead to reduction of the fracture with ligamentotaxis [42].

As in most percutaneus surgical techniques implant positioning and accuracy is controlled with fluoroscopic image intensifiers. Correct positioning of the image intensifier will lead to much lesser radiation dose for the surgeon. Placement of x-ray tube in the image intensifier on the opposite side of the surgeon will causes up to 10 times less radiation exposure [43].

#### Navigation

Balloon placement accuracy can be significantly improved and the radiation exposure during kyphoplasty can be reduced by as much as 76%, if computer-assisted fluoroscopic navigation is applied [44,45]. While relying on the navigator during the transpedicular balloon placement, balloon inflation and cement injection have to be performed under fluoroscopic control to minimise endplate fractures and cement leakage.

#### Eggshell procedure

An eggshell-procedure may avoid cement leakage in VCF suscpicious for endplate or posterior wall damage [46]. After reduction with the kyphoplasty balloon a small amount of doughy cement is injected into the cavity, and then the balloon reinserted and reinflated. Once the cement hardens the cavity can be filled with cement within the "eggshell", preventing cement leakage.

#### Choice of cement

Most vertebroplasty and kyphoplasty procedures have been performed using polymethylmethacrylate (PMMA) cement to augment the fractured vertebra. The increasing availability of injectable calcium phosphate (CaP) cement led to its application in the augmentation of compression fractures as an alternative to PMMA. Advantages are high biocompatibility, no systemic toxic monomers, osteoinductive capacity, and close to isothermal cristallinisation. Disadvantages are besides less clinical long-term experience, lesser compressive strength than PMMA [47], and the risk of early resorption, leading to defects prone to re-fractures [48-50]. The available data does not encourage the clinical use of CaP-cement in burst-fractures, flexion-distraction injuries, or rotational instable fractures [48,51].

## Results and complications of kyphoplasty

Fourteen years after the first vertebroplasty was performed in 1984, balloon kyphoplasty challenged the conventional augmentation procedures promising less complications and sagittal reconstructive ability. Until now several non-randomized prospective controlled trials have been published comparing kyphoplasty to non-surgical treatment and vertebroplasty (Table 2). Besides pain improvement and quality of life, correction of deformity and intra- and postoperative complications were investigated. The recently presented preliminary 1-year-results of the multicentrical randomized controlled Fracture Reduction Evaluation (FREE) study present in the kyphoplasty group a significant improvement of the quality of life (EQ-5D (EuroQoL), p < 0.001), pain (VAS, p < 0.0001), and function (SF-36 (Short form Health Survey), p < 0.0001, ODI, p < 0.0001) after 1 month (n = 149) controlled against non-surgical treatment (n = 151) [13]. These treatment-effects diminished dramatically until the 12-month follow-up, but were still significantly better than non-surgical treatment for quality of life as measured with EQ-5D (p < 0.05).

**Table 2:** Overview on comparative clinical trials of kyphoplasty

Author	Year	Design	Level of evidence	Control Group	Control n (levels)	Kyphoplasty n (levels)	Follow-up	Outcome
Weisskopf et al. [54]	2003	Retrospective	IIIb	non-surgical	20 (35)	22 (37)	10 days	Improvement in VAS (p < 0.001) Reduced days in hospital (p < 0.01)

Fourney et al. [55]	2003	Retrrospective	IIIb	vertebroplasty	34 (65)	15 (32)	4,5 months	No significant differences in VAS and ODI Improvement of kyphosis with kyphoplasty (p < 0.01)

Komp et al. [56]	2004	Prospective	IIb	non-surgical	19(19)	21(21)	6 months	Improvement of VAS and ODI with kyophoplasty (p < 0.01)

Kasperk et al [57]	2005	Prospective	IIb	non-surgical	20 (33)	40 (72)	12 months	Improvement of VAS (p < 0.01) and improvement of kyphosis (p < 0.001) with kyphoplasty

Grohs et al. [58]	2005	Prospective	IIb	vertebroplasty	23 (29)	28 (35)	24 months	No significant difference in ODI, but improvement of VAS with kyphoplasty (p < 0.05). No significant improvement of kyphosis

Masala et al. [59]	2005	Retrospective	IIIb	vertebroplasty	26 (33)	7 (7)	6 months	No significant difference in VAS.

Pflugmacher et al [30]	2005	Prospective	IIb	vertebroplasty	20 (32)	22 (35)	12 months	No significant difference in VAS and ODI. Improvement of kyphosis with kyphoplasty (p < 0.05)

De Negri et al. [60]	2007	Prospective	IIb	vertebroplasty	10 (18)	11 (15)	6 months	No significant difference in VAS and ODI.

Zhou et al. [61]	2008	Prospective	IIIb	vertebroplasty	42	56	12 months	No significant differences in VAS, operation time and blood loss. Improved vertebral height restoration with kyphoplasty (p < 0.01).

Wardlaw et al. [13]	2009	Randomised	Ib	non-surgical	149	151	12 months	Significant improvement in EQ-5D (p < 0.05), RMDQ (p < 0.001) VAS (p < 0.0001).

Schmelzer-Schmied et al. [51]	2009	Prospective	IIb	non-surgical	20	20	12 months	Significant greater improvement of VAS (p < 0.05) with kyphoplasty, which was lost after 3 months, and vertebral height preservation after 12 months (p < 0.01)

Schofer et al. [62]	2009	Prospective	IIIb	vertebroplasty	30	30	13 months	No significant differences in VAS and SF-36. Greater improvement of kyphotic angle with kyphoplasty (p < 0.001)

Li X et al [63]	2011	Prospective	IIIb	vertebroplasty	40	45	12 months	No significant differences in VAS and ODI. Significantly greater improvement of kyphotic angle with kyphoplasty (p < 0.01)

VAS: Visual Analogous Scale, ODI: Oswestry Disability Index, EQ-5D: EuroQoL, RMDQ: Roland Morris Disability Questionnaire, SF-36: Short Form Health Survey.

The comprehensive meta-analysis of Lee et al [52] summarized all published kyphoplasty complications. Cement leakages occurred in 14% of all cases, but only 0.01% were symptomatic. New vertebral fractures occurred in 17%. Taylor et al [53] found in their metaanalysis furthermore spinal stenosis with spinal cord compression occurred 0.16% of all cases. Radiculopathy was found in 0.17% of all cases. Furthermore there are anecdotal reports of infections after kyphoplasty [26]. The overall mortality was 4.4%, and the perioperative mortality was 0.13% [53].

## Conclusions

Kyphoplasty is - in the hands of an experienced spine surgeon or radiological interventionalist - an effective tool to treat pain caused by thoracolumbar vertebral compression fractures, but the severity of pulmonary PMMA cement embolism and the urgent need of immediate decompression in relevant spinal stenosis after cement leakage, require an anaesthesiologist and a spinal surgeon on call. The complication rate of kyphoplasty is dramatically lower than in alternative open instrumented procedures, and the immediate pain reduction is significantly greater in kyphoplasty compared to conservative treatment. Therefore its application remains a pillar in VCF treatment.

## Competing interests

YR, CEH, and PF were clinical investigators in the FREE trial, and YR and PF were Clinical Investigators in the CAFE trial, both initiated by Kyphon Inc. (now Medtronic Spine LLC, Sunnyvale, CA, USA). YR, CEH, PF and CO received travel assistance by Medtronic, DePuy Spine (Johnson & Johnson) and Synthes.

## Authors' contributions

YR wrote the manuscript, and CEH, PF and CO revised it critically. All authors read and approved the final manuscript.
